# Peripheral Blood Serum NMR Metabolomics Is a Powerful Tool to Discriminate Benign and Malignant Ovarian Tumors

**DOI:** 10.3390/metabo13090989

**Published:** 2023-09-01

**Authors:** Sofia C. Nunes, Joana Sousa, Fernanda Silva, Margarida Silveira, António Guimarães, Jacinta Serpa, Ana Félix, Luís G. Gonçalves

**Affiliations:** 1 iNOVA4Health, NOVA Medical School, Faculdade de Ciências Médicas, NMS, FCM, Universidade NOVA de Lisboa, Campo dos Mártires da Pátria 130, 1169-056 Lisboa, Portugal; asofianunes5@gmail.com (S.C.N.); jacinta.serpa@nms.unl.pt (J.S.); ana.felix@nms.unl.pt (A.F.); 2 Instituto Português de Oncologia de Lisboa Francisco Gentil (IPOLFG), Rua Prof Lima Basto, 1099-023 Lisbon, Portugal; 3 Instituto de Tecnologia Química e Biológica António Xavier (ITQB NOVA), Avenida da República (EAN), 2780-157 Oeiras, Portugal

**Keywords:** NMR metabolomics, ovarian cancer, benign ovarian tumors, malignant ovarian tumors, borderline ovarian tumors, biomarkers, cancer progression, serum

## Abstract

Ovarian cancer is the major cause of death from gynecological cancer and the third most common gynecological malignancy worldwide. Despite a slight improvement in the overall survival of ovarian carcinoma patients in recent decades, the cure rate has not improved. This is mainly due to late diagnosis and resistance to therapy. It is therefore urgent to develop effective methods for early detection and prognosis. We hypothesized that, besides being able to distinguish serum samples of patients with ovarian cancer from those of patients with benign ovarian tumors, ^1^H-NMR metabolomics analysis might be able to predict the malignant potential of tumors. For this, serum ^1^H-NMR metabolomics analyses were performed, including patients with malignant, benign and borderline ovarian tumors. The serum metabolic profiles were analyzed by multivariate statistical analysis, including principal component analysis (PCA) and orthogonal partial least squares discriminant analysis (OPLS-DA) methods. A metabolic profile associated with ovarian malignant tumors was defined, in which lactate, 3-hydroxybutyrate and acetone were increased and acetate, histidine, valine and methanol were decreased. Our data support the use of ^1^H-NMR metabolomics analysis as a screening method for ovarian cancer detection and might be useful for predicting the malignant potential of borderline tumors.

## 1. Introduction

Ovarian cancer includes a group of complex and distinct diseases [1], is a major cause of death from gynecological cancer and is the third most common gynecological malignancy worldwide [2]. There are several known risk factors for ovarian cancer, including genetic, physiological, socioeconomic and environmental aspects [3].

Ovarian tumors can develop from epithelial cells, stromal cells and germ cells [4]. Carcinomas (i.e., tumors originated from epithelial cells) include more than 90% of ovarian malignancies [4,5] and can be classified based on histopathology and molecular/genetic features, being mainly classified as serous low-grade and high-grade, endometrioid, clear cell, Brenner tumors and mucinous [4,5,6]. These different histological types are associated with different clinical outcomes, including the response to treatment and the prognosis of the disease [7].

Borderline tumors include a subgroup of epithelial- and stromal-derived ovarian tumors with unknown prognosis. However, evidence suggest that malignant progression can occur, mainly in the serous type. The majority of stromal-derived tumors are granulosa and Sertoli–Leydig cell tumors, all with different biologic, pathogenetic and molecular features [8]. Germ cell tumors are frequent but usually benign [4].

In the last 30 years there has been a slight improvement in the overall survival of patients with ovarian carcinoma; however, the cure rate did not improve [9]. This is mainly due to late diagnosis and resistance to therapy [5,10]. In fact, ovarian carcinoma is generally diagnosed in advanced stages due to the absence of clear symptoms in the early stages of the disease [11,12]. It is therefore urgent to develop effective early detection methods. In addition, albeit generally presenting an excellent prognosis, borderline tumors can recur as well as undergo malignant progression [8,13]; thus, it is also urgent to find methods to predict the prognosis of these tumors. This would ultimately allow close follow-up of these patients, possibly resulting in an improvement of their outcome.

Growing evidence suggests serum metabolomics analysis as a promising tool for diagnosis, prognosis and treatment response prediction in several types of cancer [14,15,16,17,18,19,20,21,22,23]. Specifically, ^1^H-NMR metabolomics analysis provides several advantages over other methods, given the simplicity of sample preparation, the very high reproducibility, its non-destructive nature, and the detection and quantification of several metabolites simultaneously in one single measurement [24,25]. In the specific context of ovarian cancer, some reports have already shown that peripheral blood serum metabolomics through ^1^H-NMR analysis can be a promising tool for the discovery of new biomarkers for the early detection of the disease. In 2005, Odunsi and colleagues have reported that ^1^H-NMR metabolomics analysis of serum is able to separate epithelial ovarian cancer patients from both premenopausal normal samples and from patients with benign ovarian disease [26]. In 2011, Garcia and co-workers reported that serum ^1^H-NMR-based metabolomics allows the early-stage diagnosis of epithelial ovarian cancer [27]. ^1^H-NMR metabolomics analysis can also be a valuable tool in the understanding of disease progression. More recently, Bharti et al., aimed to characterize the metabolic composition of malignant ascites derived from orthotopic growth of two different ovarian cancer cell lines [28]. Thus, the authors have used high-resolution ^1^H-NMR analysis, and, albeit finding different metabolite patterns in ascitic fluid derived from OVCAR3 and ID8-VEGF-Defb29 tumor-bearing mice, the authors also found common metabolites, including β-hydroxybutyrate, maleic acid and citrate [28]. The authors have then suggested the metabolic characterization of malignant ascites as a powerful method providing new insights into the role of ascitic fluid in supporting cancer cell growth and resistance to treatment [28].

In this study, we have hypothesized that besides being able to separate serum samples of patients with ovarian cancer from patients with benign ovarian tumors, ^1^H-NMR metabolomics analysis might predict the malignant potential of borderline tumors. For this, serum ^1^H-NMR metabolomics analysis was performed, including 41 peripheral blood serum samples from patients with ovarian cancer, 45 from patients with ovarian benign neoplasms and 9 from patients with ovarian borderline tumors. The samples were derived from patients with ovarian neoplasms diagnosed, mainly of epithelial origin, but samples from patients with stromal and germ cells tumors were also included. The serum metabolic profiles were analyzed by multivariate statistical analysis, including principal component analysis (PCA) and orthogonal partial least squares discriminant analysis (OPLS-DA) methods.

## 2. Methods

### 2.1. Ethics Statement

This study was reviewed and approved by the ethical committee of the Instituto Português de Oncologia de Lisboa, Francisco Gentil (Reference UIC/1080).

### 2.2. Sample Collection

Peripheral blood from patients with benign, malignant and borderline ovarian neoplasms was collected and allowed to clot at room temperature for 15–30 min. The clot was then removed by centrifugation at 956× *g* for 5 min at 4 °C. The resulting supernatant was stored at −80 °C until NMR analysis.

### 2.3. NMR Spectral Acquisition, Processing and Metabolite Identification

Prior to NMR analysis, the serum samples were thawed at room temperature and 300 μL of serum were added 300 μL of a sodium phosphate buffer solution prepared in 80% H_2_O and 20% D_2_O, and 560 μL were transferred to a 5 mm NMR tube.

Serum samples were analyzed at 300 K in a 600.10 MHz Bruker Avance III spectrometer, equipped with a 5 mm four channel cryo-probe (QCI-Z H/C/P/N/-D) and a refrigerated autosampler. From each sample, a 1D ^1^H-NMR spectrum with a noesy1d pulse sequence and water suppression (noesygppr1d) as a sum of 32 free induction decays (FID), with 64 K complex points (TD), using a spectral window of 30 ppm (18,028.846 Hz), 4 s relaxation delay and 10 ms of mixing time; a 1D ^1^H-NMR spectrum using a CPMG pulse sequence with water presaturation (cpmgpr1d) as a sum of 32 FIDs, with 64 k TD, using an SW of 20 ppm (12,019.23 Hz), 4 s relaxation delay, an echo time of 0.3 ms and 126 repetitions of the echo time per scan; and a 1D ^1^H-NMR spectrum diffusion-edited pulse program using a presaturation (ledbpgppr2s1d) as a sum of 32 FIDS, with 64 k TD, using an SW of 30 ppm (18,028.846 Hz), 4 s relaxation delay and a diffusion time of 0.3 ms was acquired.

Spectral processing was carried out using the TopSpin software version 3.2 (Bruker BioSpin, Rheinstetten, Germany). The chemical shifts were referenced internally to the anomeric proton signal of α-d-glucose at 5.22 ppm. To aid metabolite identification, two-dimensional NMR spectroscopy experiments were acquired: 2D ^1^H J-resolved spectra were acquired for each sample and homonuclear (^1^H–^1^H) total correlation spectroscopy (TOCSY), (^1^H–^13^C) hetero-nuclear single quantum correlation spectroscopy (HSQC) were carried out for selected samples. Metabolite identification was performed by comparison with NMR spectra from pure standards available in the Human Metabolome Database (HMDB) [29].

### 2.4. Statistical Data Analysis

The processed CPMG and LED 1D ^1^H-NMR spectra were transformed into a matrix (each column was a spectrum; each row was one of the 128 k points that makes up the FID). The water region was removed from the spectra (4.70–5.0) and the spectra aligned, for the analysis only the region between 0.15–10.00 ppm was used. This spectra processing was performed in the R software environment for statistical computing (version 3.3.2) using in-house scripts [25]. The multivariate analysis of the spectra principal component analysis (PCA), partial least squares discriminant analysis (PLS-DA) and orthogonal partial least squares discriminant analysis (OPLS-DA) were performed using SIMCA 13.0.3. A cross-validation method was used to evaluate the prediction quality (Q2) value of the resulting models; a 999 permutation analysis was applied to confirm the PLS-DA model validity [30]. The predictive ability of the OPLS-DA models was assessed by predicting 7 subsets of the dataset on the model created with the remaining data (sevenfold cross-validation). Ten samples of each group, benign and malignant, were removed from the analysis and used as a validation set. The OPLS-DA model was used to predict the classification of the 9 borderline samples. The spectral variables responsible for the differences between groups were identified from the loading weights and variable importance to the projection (VIP) values of the cross-validated OPLS-DA models.

The differences in metabolites were further evaluated using Mann–Whitney–Wilcoxon non-parametric tests on the spectral areas of the corresponding metabolite peaks after local baseline correction; the obtained *p*-values were false discovery rate (FDR)-adjusted (q-values) [31]. The area values of these metabolites were used to perform metabolic pathway analysis in MetaboAnalyst 4.0 (https://www.metaboanalyst.ca/faces/home.xhtml, accessed on 2 October 2020).

## 3. Results

### 3.1. Study Population

This study included 95 patients with ovarian neoplasms, from which 45 were benign, 41 were malignant, and 9 were borderline neoplasms. Regarding tumor origin, 69 patients were diagnosed with epithelial cell-derived neoplasms, 12 tumors derived from stromal cells, 7 from germ cells, and 7 were of other origin (Table 1).

### 3.2. ^1^H-NMR Spectra Separate Serum from Patients with Ovarian Cancer from Patients with Benign Neoplasms

**Figure 1:**
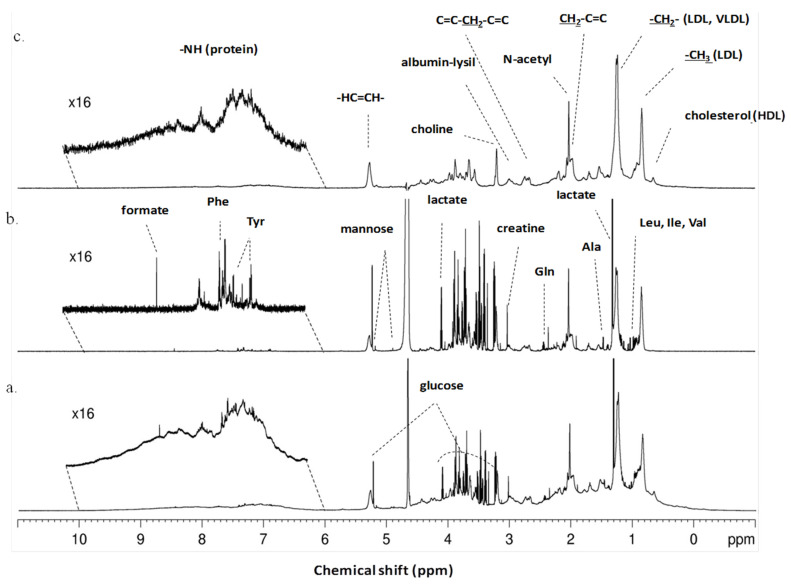
Typical ^1^H-NMR serum spectra of patients with ovarian neoplasms. (**a**) Typical ^1^H-NMR serum spectra (pulse sequence = noesygppr1d), (**b**) Typical CPMG ^1^H-NMR serum spectra showing only small molecules and (pulse sequence = cpmgpr1d) (**c**) Typical LED ^1^H-NMR serum spectra showing only large molecules (pulse sequence = ledbpgppr2s1d). Some of metabolites and functional groups detected are indicated.

We started by identifying the metabolites present in serum derived from patients with malignant and benign ovarian neoplasms. The typical ^1^H-NMR spectra of serum from patients with ovarian neoplasms are shown in Figure 1. The utilization of different NMR pulse programs allows the extraction of different information about the serum: with the CPMG experiments we obtain information about the metabolites (Figure 1b), while with the LED spectra we obtain information about the macromolecules present in the serum (Figure 1c). The qualitative analysis of the spectra allows the identification of several different metabolites, including several amino acids, lipids, organic acids and glucose, which are present in serum derived from patients with both benign and malignant ovarian neoplasms (Figure 1).

To evaluate the differences between benign and malignant neoplasms, serum ^1^H-NMR spectra obtained with CPMG and LED pulse programs were analyzed through PCA. This analysis was able to separate serum samples from patients with benign neoplasms from patients with malignant neoplasms, therefore reflecting significant differences in terms of blood serum composition between groups (Figure S1).

**Figure 2:**
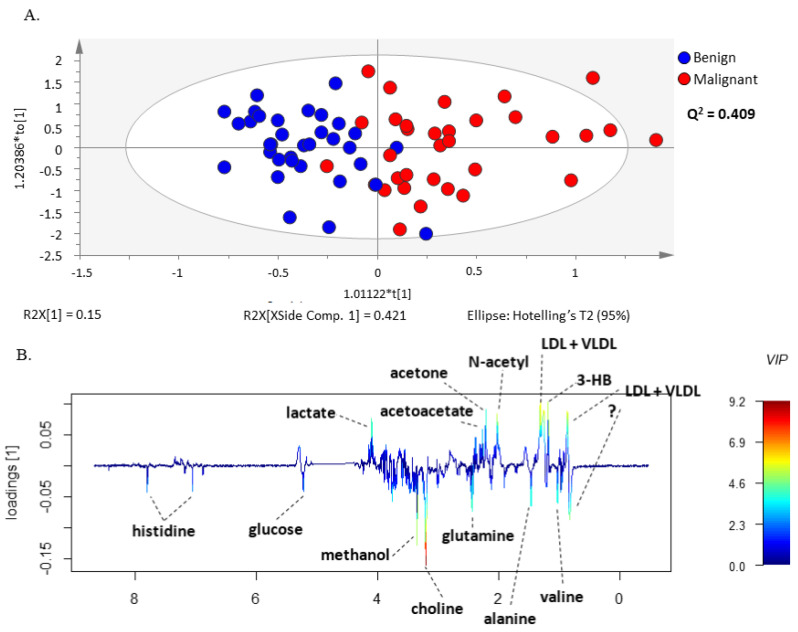
OPLS-DA model of CPMG spectra reveals different sets of metabolites increased in serum from patients with ovarian cancer compared to benign neoplasms. (**A**) OPLS-DA scores scatter plot of CPMG spectra (small molecules) and (**B**) Loading weights line plot of Component 1 color-coded with the corresponding variable importance to the model (VIP value).

**Figure 3:**
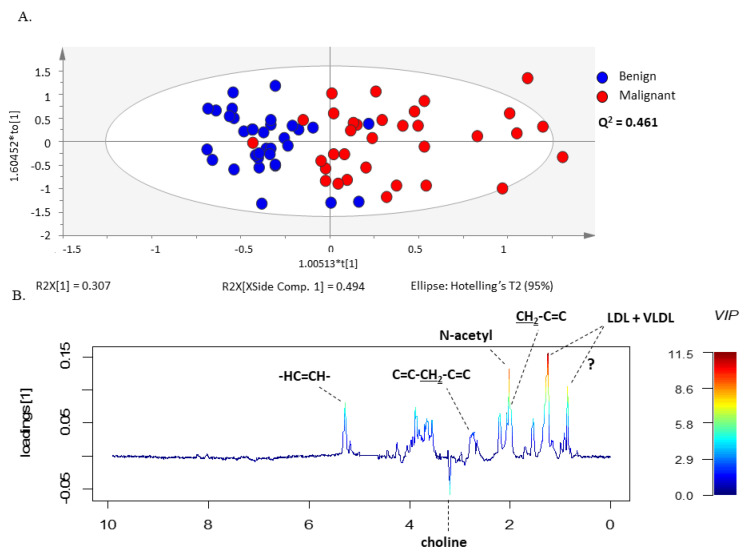
OPLS-DA model of LED spectra reveals different sets of metabolites increased in serum from patients with ovarian cancer compared to benign neoplasm. (**A**) OPLS-DA scores scatter plot of LED spectra (large molecules) and (**B**) Loading weights line plot of Component 1 color-coded with the corresponding VIP value.

The same set of benign and malignant neoplasm serum spectra was further examined by the supervised multivariate statistical method, PLS-DA (Figure S2) and OPLS-DA, analyzing again the CPMG (Figure 2A) and the LED (Figure 3A) spectra separately in the two groups. The models constructed by these methods made it possible to distinguish between serum samples from patients with malignant and benign ovarian tumors. In addition, these methods allowed the construction of a predictive multivariate model based on malignant neoplasms serum ^1^H-NMR spectra, also enabling the identification of the metabolites with greatest impact on the discrimination between groups. The robustness of the OPLS-DA model was evaluated by cross-validation. By using a tenfold cross-validation procedure, the model presented a prediction quality parameter (Q^2^) of 0.409 for small molecules and 0.461 for large molecules, respectively. Further validation was performed by CV-ANOVA, analysis of variance testing of Cross-Validated predictive residuals in the case of the OPLS-DA model and by permutation analysis for the PLS-DA model (Table S1 and Figure S3).

The validated OPLS-DA model enabled identification of the metabolite resonances responsible for the separation of serum samples from the two groups of patients through the analysis of the model weight loadings and VIP parameter. Only the signals corresponding to VIP values > 1 were considered as having a significant impact on group separation. This criterion was fulfilled for histidine, glucose, methanol, choline, glutamine, alanine, valine, lactate, acetoacetate, acetone, LDL + VLDL, 3-hydrobutyrate, N-acetyl functional group and lipids functional groups (Figure 2 and Figure 3). Interestingly, the corresponding loading weights revealed that several metabolites were differently increased in serum from patients with malignant tumors compared to benign neoplasms. Therefore, an increase in histidine, glucose, methanol, choline, glutamine, alanine and valine was found in serum from patients with benign neoplasms, whereas patients with malignant tumors presented increased serum values in lactate, acetoacetate, acetone, LDL + VLDL, 3-hydrobutyrate as well as N-acetyl functional group and lipids functional groups (Figure 2B and Figure 3B).

### 3.3. Lactate, 3-Hydroxybutyrate, Acetone, Acetate, Histidine, Valine and Methanol Separate Serum from Patients with Ovarian Cancer from Patients with Benign Neoplasm

**Figure 4:**
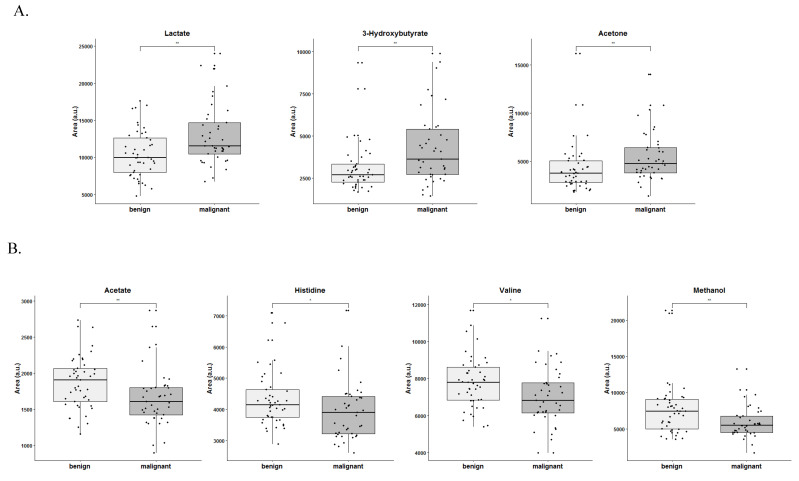
Different sets of metabolites are increased in serum from patients with ovarian cancer compared to benign neoplasms. (**A**) Metabolites increased in serum from patients with malignant ovarian tumors and (**B**) Metabolites increased in serum from patients with benign ovarian neoplasms. * *p* < 0.05, ** *p* < 0.01 (non-parametric univariate tests adjusted for multiple testing).

The differences in metabolites between groups were further assessed by comparing the corresponding spectral areas using non-parametric univariate tests adjusted for multiple testing. Therefore, lactate, 3-hydroxybutyrate, acetone, acetate, histidine, valine and methanol were significantly altered between groups, in which lactate, 3-hydroxybutyrate and acetone were significantly increased in serum samples from patients with malignant tumors (Figure 4A), whereas acetate, histidine, valine and methanol were decreased in serum samples from patients with malignant tumors (Figure 4B). Given the impossibility of correctly assessing the spectral areas, this analysis was not performed for some of the metabolites such as choline.

**Figure 5:**
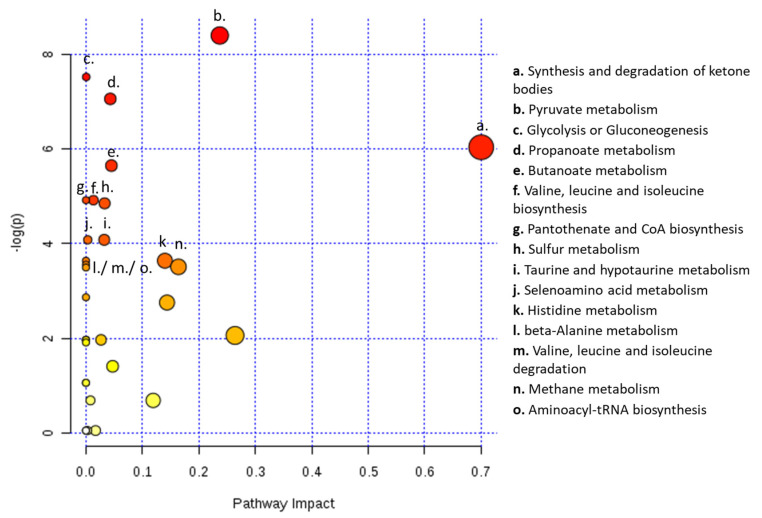
Serum from ovarian cancer patients shows alterations in several metabolic pathways. Metabolic pathway analysis for NMR metabolomics analysis of serum from patients with ovarian cancer and with ovarian benign neoplasms. All the matched pathways are displayed as circles. The color and size of each circle are based on *p*-value and pathway impact value, respectively. The most impacted pathways having statistical significance (*p* < 0.05) are indicated.

Moreover, metabolic pathway analysis predicted 16 metabolic pathways significantly and differently altered in serum from patients with ovarian cancer compared to patients with benign neoplasms. Therefore, the analysis showed alterations in the following pathways: synthesis and degradation of ketone bodies; pyruvate metabolism; glycolysis and/or gluconeogenesis; propanoate metabolism; butanoate metabolism; valine, leucine and isoleucine biosynthesis; pantothenate and CoA biosynthesis; sulfur metabolism; taurine and hypotaurine metabolism; selenoamino acid metabolism; histidine metabolism; beta-alanine metabolism; valine, leucine and isoleucine degradation; methane metabolism and aminoacyl-tRNA biosynthesis (Figure 5).

### 3.4. OPLS-DA Model Predicted the Outcome of Bordeline Tumors

These OPLS-DA models were applied to predict the class, malignant or benign, of the serum samples of the borderline ovarian tumors patients. From the nine patients, six were assigned as belonging to the benign class while three were assigned to the malignant class (Table 2). The same result was obtained for both the CPMG-based model and the LED-based model.

## 4. Discussion

Ovarian cancer is a highly lethal disease [11] that is normally diagnosed in advanced stages, where a cure is difficult due to chemoresistance and recurrence of the disease [32,33]. The absence of clear symptoms and the non-existence of effective early-screening methods hinder the improvement of EOC outcomes; thus, it is urgent to find novel effective biomarkers allowing the early detection and prediction of disease progression.

In this study, we were able to separate patients with ovarian cancer from those with benign tumors by means of serum ^1^H-NMR metabolomics analysis, hence strengthening the role of metabolomics in the screening of ovarian cancer. Importantly, our model has identified lactate, 3-hydroxybutyrate, acetone, acetate, histidine, valine and methanol as discriminant metabolites between serum derived from patients with benign or malignant ovarian tumors.

We observed increased levels of lactate in serum from ovarian cancer patients compared to serum from patients with benign ovarian tumors. Lactate is mainly generated by cancer cells through increased glycolysis rate to support the biosynthesis needed to sustain cancer cell proliferation, as recently reviewed by Serpa [34]; it is expected that the levels of lactate directly correlate with cancer features, accounting for a faster disease progression. However, some controversy remains. While some studies [35,36,37] on ovarian cancer patients and models have reported increased levels of lactate, decreased lactate levels were also found in serum and urine samples from patients with ovarian cancer compared to control groups [38]. Regarding lactate, our results are concordant with the data of Kyriakides et al., as we observed higher lactate levels in serum from patients with malignant tumors compared to benign ones. However, our data support increased choline levels in serum derived from patients with benign neoplasms compared to malignant ones. We must highlight that, besides different biological samples, the authors only included ovarian epithelial tumors, which could explain the contradictory results. Additionally, the decreased levels of lactate reported in some studies can be explained by the increased levels of lactate dehydrogenase (LDH) detected in ovarian cancer patients’ serum compared to serum from individuals with benign neoplasms [39]. Nevertheless, the levels of LDH in blood serum of ovarian cancer patients depends on the histological type [40,41] and tumor stage, and is proposed as a marker of poor prognosis [42]. Moreover, most studies on serum samples comprised ovarian high-grade serous patients only [43], whereas our study comprises several histological types of ovarian cancer, which could explain the different results obtained. Therefore, the detected lactate levels are influenced by the LDH activity in the blood serum of patients, and, because most scientific papers dedicated to this issue were published more than 30 years ago, further research is needed.

Lipids are important nutrients for cancer cells, whose degradation can originate ketone bodies through ketogenesis [44]. In turn, ketone bodies are thought to be used by certain cancer cells as fuel [45,46], and increased 3-hydroxybutyrate was found in a study dedicated to endometrial cancer [47]. Interestingly, the increased levels of the ketone body 3-hydroxybutyrate observed in our study have also been linked in other studies to lipolysis as an alternative source of energy production by the malignant cells via upregulation of fatty acid oxidation [27,43]. Serum 3-hydroxybutyrate and acetone were previously found to be elevated in patients with early stage EOC compared to healthy controls [27]. Interestingly, metabolites of hydroxybutyric acid were reported as novel diagnostic and prognostic biomarkers of ovarian high-grade serous carcinomas, including serum 3-hydroxybutyric acid [43]. Odunsi and colleagues also reported increased serum 3-hydroxybutyrate levels in EOC patients compared both to pre- and postmenopausal controls [26].

In our study, we found an association between ovarian cancer and decreased levels of acetate, valine, histidine and methanol. Acetate can be used by cancer cells as a bioenergetic or biosynthetic source. Decreased acetate and valine levels were previously reported in urine samples from ovarian cancer patients when compared to healthy individuals [48]. Acetate is a pivotal compound in lipids metabolism, since acetyl-CoA can be generated from acetate resulting from fatty acids oxidation (degradation) and be deviated to supply the TCA cycle and energy production or redundantly be incorporated in fatty acids synthesis [49]. Thus, in serum from ovarian cancer patients we confirmed that lipids oxidation occurs, because the levels of 3-hydroxybutyrate were augmented and the acetate levels were diminished, suggesting that lipids play an important role in ovarian cancer metabolic remodeling. Niemi and colleagues reported changes in lipid metabolism in early-stage ovarian cancer that increases with the stage of the disease, reporting also these lipidomic changes in different histological subtypes of ovarian cancer, including high-grade serous, mucinous and endometrioid ovarian carcinomas [50].

In accordance with our findings, Garcia and colleagues also reported decreased serum valine levels in serum samples from early-stage ovarian cancer patients when compared to healthy individuals [27]. Valine is a branched amino acid with pivotal roles in cancer biology. Besides being a component of proteins, valine is also an important source of organic compounds that can be used to supply the TCA cycle, namely the succinyl-CoA [51], being further used in energy or biomass production. In endometrial cancer, valine was considered an important marker to distinguish blood serum from women with endometrial carcinoma and healthy women [47].

Decreased plasma histidine concentrations in patients with ovarian cancer were also reported by other groups in which patients with EOC were found to present lower levels of histidine compared to control groups [52,53,54]. Decreased histidine [55] and methanol [48] levels were also reported in urine samples from patients with ovarian cancer compared to healthy controls [55]. Controversially, Plewa and colleagues have pointed out histidine and citrulline as putative ovarian cancer biomarkers and also reported lipid compounds as new possible biomarkers of ovarian cancer [54]. The biological impact of histine in cancer biology is under scrutiny and some studies have linked histidine catabolism as a way of sensitizing cancer cells to methotrexate, a folate antagonist used in cancer chemotherapy [56,57]. This shows that histidine is very important in one-carbon metabolism, composed by the folate cycle and methionine cycle, crucial not only in the metabolic network functioning but also in epigenetic regulation [58].

It is important to highlight that ovarian cancer comprises distinct diseases that have a common anatomical location [1], and in this research we did not separate ovarian cancer patients by the histological type of the disease, nor by its stage or cellular origin, since we aimed to find a malignancy metabolic signature for ovarian cancer. In fact, cancer features could explain the high diversity of biomarkers found for ovarian cancer until now, as well as the different analytical techniques that were used. For instance, Chen and colleagues have performed metabolomics analysis via mass spectrometry and, besides being able to separate healthy women from patients with benign and malignant ovarian neoplasms, the authors also suggested the metabolite 27-nor-5β-cholestane-3,7,12,24,25 pentol glucuronide as a new biomarker for epithelial ovarian cancer [59]. Importantly, the authors found this compound upregulated in the early stage of disease and in serous, endometrioid and mucinous carcinomas but not in clear cell carcinoma [59]. Sellem and coworkers, by using ^1^H HRMAS NMR spectroscopy, found different metabolic patterns among mucinous, endometrioid and serous carcinomas, where a metabolic signature specific to serous (N-acetyl-aspartate) and mucinous (N-acetyl-lysine) carcinomas was found [60]. Importantly, the authors were able to predict both survival rates and response to chemotherapy in patients with serous carcinomas [60]. Gard et al., by combining ^1^H-NMR and LC-MS metabolomics were able to separate tissue derived from high-grade and low-grade EOC from controls, also reporting altered ascorbate and aldarate metabolism in EOC patients [61]. Very recently, Zeleznik and colleagues have reported plasma pseudouridine as a putative novel risk factor of ovarian cancer in general and also in non-serous tumors [62]. The authors also found several metabolite groups and metabolite modules associated with the risk of ovarian cancer, both independent of histological type as well as histological type-specific [62]. These reports strongly support the existence of biomarkers that are both specific to and independent of the histological type of EOC.

Ke and coworkers, by liquid chromatography mass spectrometry, analyzed plasma metabolic changes related to advanced EOC at diagnosis, surgery and relapse and have identified different metabolic features and different putative biomarkers according to patient group [63]. This study strongly suggests that ovarian cancer biomarkers also differ, along with disease progression. More recently, other putative EOC biomarkers were reported, such as 2-piperidinone and 1-heptadecanoylglycerophosphoethanolamine [64] and long-chain ceramides and sphingomyelins [65].

It should be mentioned that our statistical model might correctly predict the malignant potential of tumors, hence allowing the prediction of disease progression in the case of patients with borderline tumors, which could improve the outcome of these patients by facilitating their follow-up. In 2006, Denkert and colleagues, by gas chromatography/time-of-flight mass spectrometry, were able to separate ovarian borderline tumors from invasive carcinomas using fresh frozen tumor samples [66]. Saleem and colleagues, by HRMAS-NMR analysis of epithelial ovarian tumor biopsies were able to predict borderline tumors that presented an intermediate metabolic pattern similar to the normal ovarian tissue [60]. Kyriakides et al., by ^1^H-NMR spectroscopy, have characterized the metabolic profiles of ovarian cyst fluid samples from benign, borderline and malignant ovarian tumors and reported progressively increasing levels of choline and lactate from benign to borderline to malignant samples [36]. In our evaluation setting, the percentage of borderline tumors presenting a metabolic pattern close to benign or malignant tumors was 33.3% and 66.6%, respectively. However, the borderline ovarian tumors included in this study are still surviving in a disease-free state. Thus, our model must be applied to more cases with a longer prospective follow-up time to ensure its feasibility as a predictor of the malignant transformation of borderline tumors.

Collectively, our data strengthens the power of metabolomics in ovarian cancer screening. The plausible use of the different metabolites reported here as putative biomarkers should be further explored. As differences in metabolic patterns among the histologic types and stages of ovarian cancer were reported, common metabolic patterns were also found regarding the different histological types of ovarian cancer. Remarkably, our metabolomics analysis reflected alterations in 16 different metabolic pathways, including energy, lipid, amino acids and ketone body metabolism, being in accordance with the metabolic reprogramming characteristic of cancer [67,68]. Therefore, our data coincides with other reports, strengthening the case for the metabolites reported in here as putative biomarkers for ovarian cancer, especially the 3-hydroxybutirate and histidine dynamics that have been consistently reported.

## 5. Conclusions

Our data support the use of ^1^H-NMR metabolomics analysis as a screening method for ovarian cancer detection, in which lactate, 3-hydroxybutyrate, acetone, acetate, histidine, valine and methanol are proposed as new putative biomarkers for this disease. Moreover, our OPLS-DA model may be useful for predicting the malignant potential of borderline tumors.

## Figures and Tables

**Table 1:** Characteristics of the included patients.

	Benign	Malignant	Borderline
Number of patients, N (%)	45 (47.4)	41 (43.1)	9 (9.5)
Age, mean ± SD, years	48.5 ± 17.2	60.1 ± 12.4	50.8 ± 26.0
CA125, mean ± SD, U/mL	43.5 ± 58	1980.7 ± 4351.4	151.1 ± 141.9
Tumor origin			
Epithelial cells (n = 69), n (%)	25 (55.6)	38 (92.7)	6 (66.7)
Stromal cells (n = 12), n (%)	10 (22.2)	-	2 (22.2)
Germ cells (n = 7), n (%)	7 (15.6)	-	-
Other (n = 7), n (%)	3 (6.7)	3 (7.3)	1 (11.1)

N, number of patients; SD, Standard deviation.

**Table 2:** Prediction of borderline tumor serum samples based on CPMG and LED OPLS-DA models. In bold are indicated the groups attribute to each samples by the OPLS-DA models.

	CPMG		LED	
Sample	P (Benign)	P (Malignant)	P (Benign)	P (Malignant)
**1**	0.19	**0.81**	−0.25	**1.25**
**2**	0.14	**0.86**	−0.59	**1.59**
**3**	**0.99**	0.01	**0.93**	0.07
**4**	0.18	**0.80**	0.45	**0.55**
**5**	0.27	**0.72**	−0.06	**1.06**
**6**	**0.93**	0.07	**0.88**	0.12
**7**	−0.52	**1.52**	−0.49	**1.49**
**8**	−0.46	**1.46**	−0.92	**1.92**
**9**	**0.79**	0.21	**0.89**	0.11

## Data Availability

The metabolomic data is deposited at Metabolights repository with the code MTBLS2124.

## Supplementary Materials

The following supporting information can be downloaded at: https://www.mdpi.com/article/10.3390/metabo13090989/s1, Figure S1: PCA models of CPMG (A) and LED spectra (B) allow to observe a separation of the serum of benign and malignant ovary tumor patients; Figure S2: PLS-DA models of CPMG (A) and LED spectra (B) allow the discrimination of the serum of benign and malignant ovary tumor patients; Figure S3: The PLS-DA models are valid on the permutation tests, CPMG (A) and LED (B); Table S1: Table with the parameters of the CV-ANOVA, ANalysis Of VAriance testing of Cross-Validated predictive residuals for the OPLS-DA models construct with CPMG and LED spectra.
