# Supplementary material for: Peripheral Blood Serum NMR Metabolomics Is a Powerful Tool to Discriminate Benign and Malignant Ovarian Tumors
Source: Metabolites. 2023 Sep 1;13(9):989. doi: 10.3390/metabo13090989 (PMC10537270; doi:10.3390/metabo13090989)
Supplement: Supplementary file 1 [file metabolites-13-00989-s001.zip › metabolites-2538923-supplementary.pdf]

Supplementary Figure S1

A

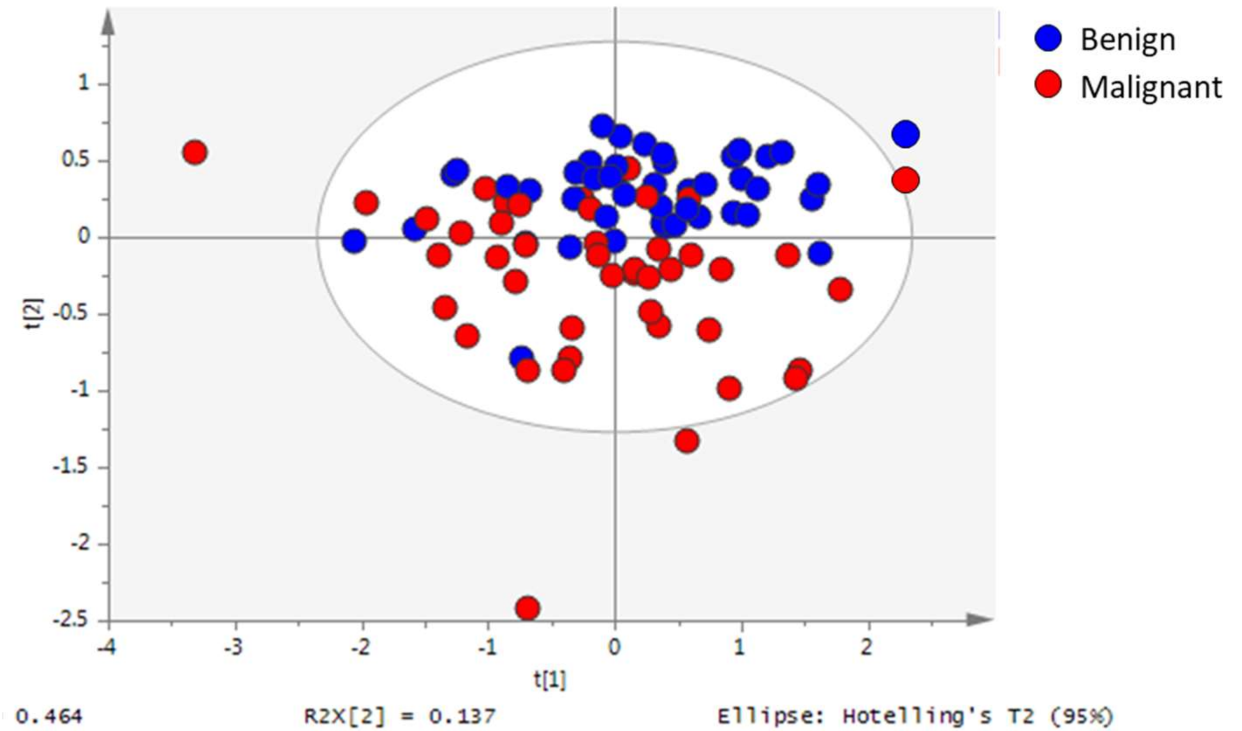

B

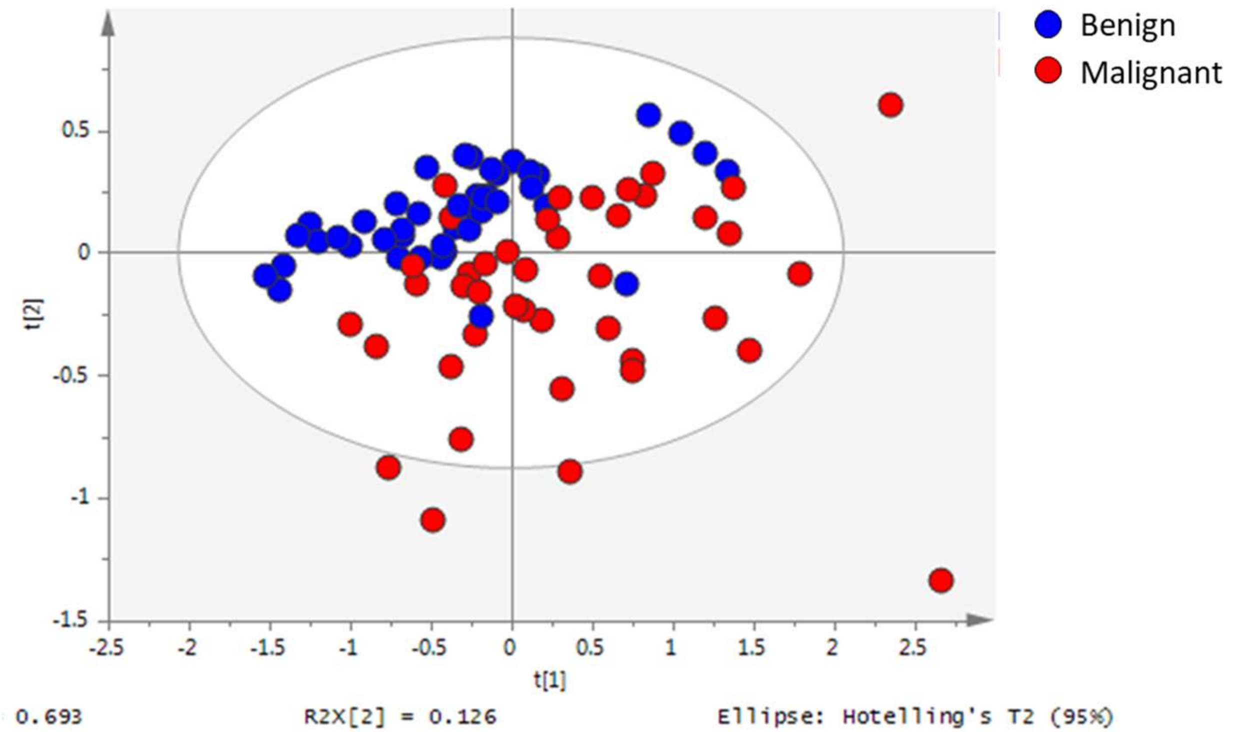

Supplementary Figure S2

A.

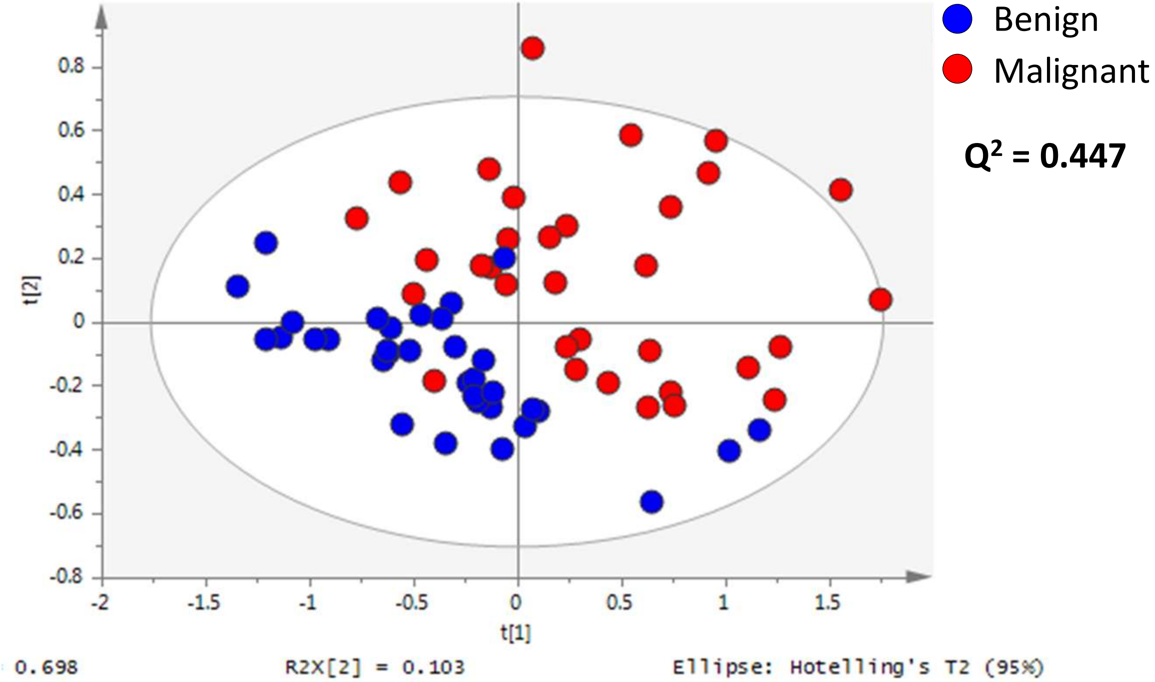

B.

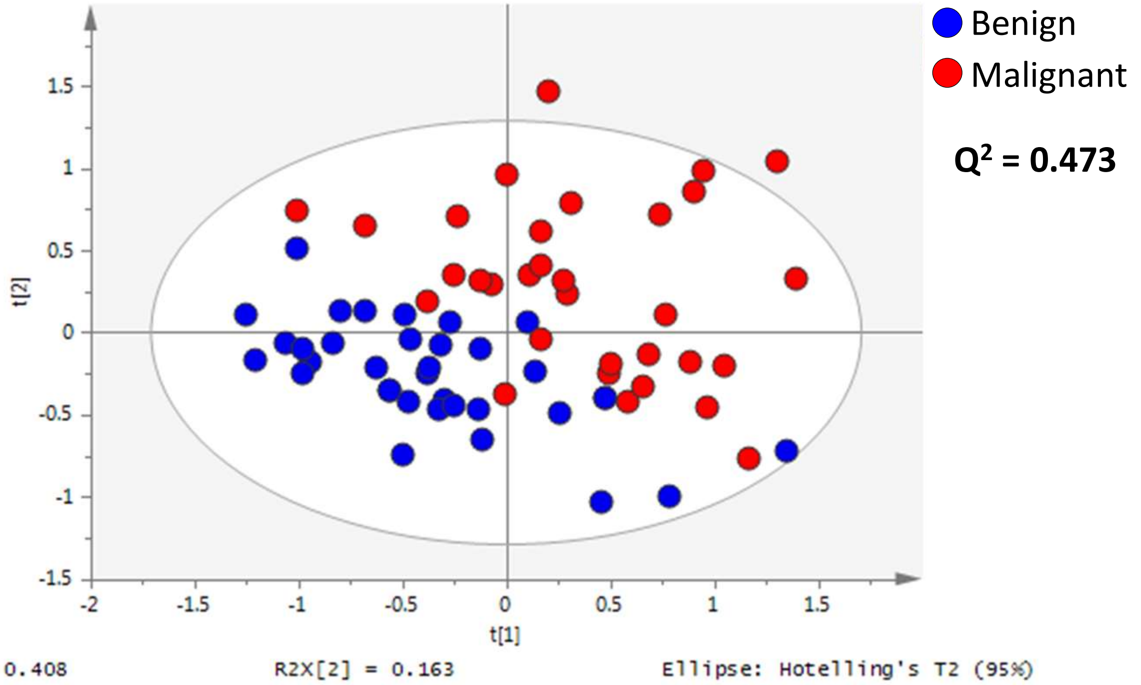

Supplementary Figure S3

A.

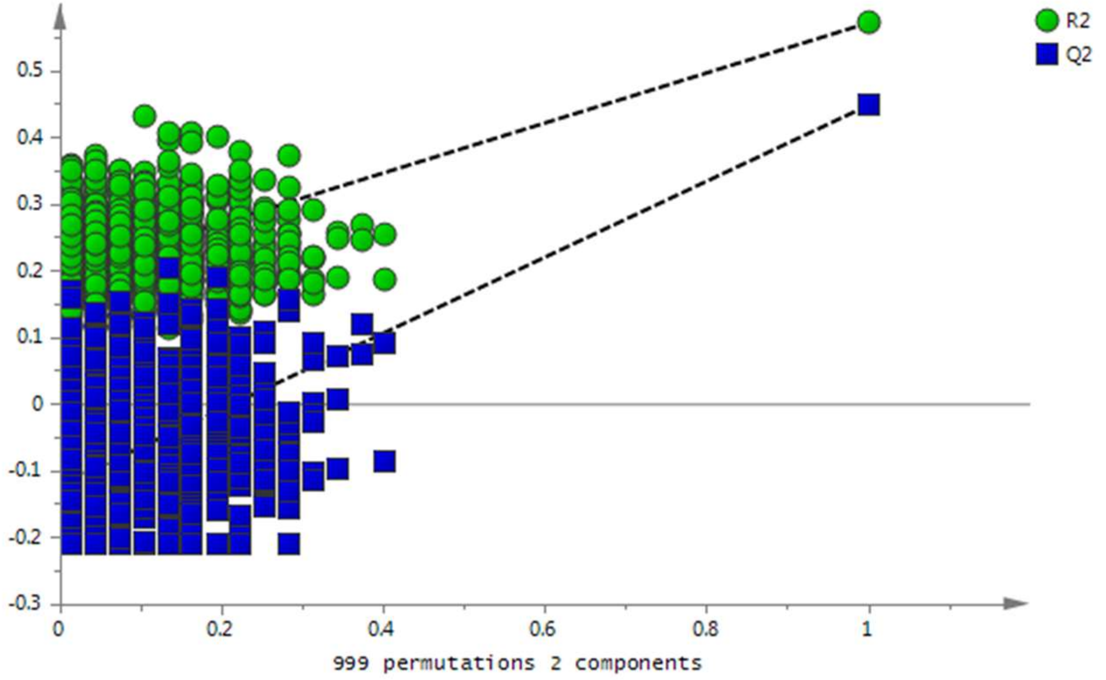

B.

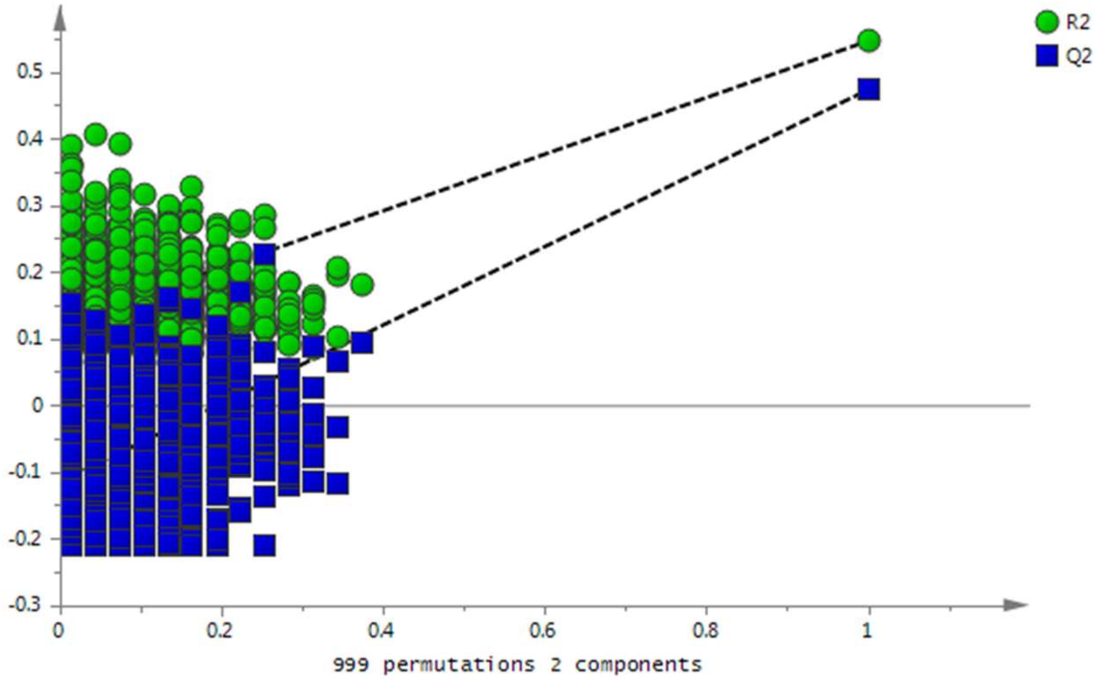

Supplementary table S1- Table with the parameters of the CV-ANOVA, ANalysis Of VAriance testing of Cross-Validated predictive residuals for the OPLS-DA models cosntruct with *cpmg* and *led* spectra.

2.	3.	4. **SS**	5. **DF**	6. **MS**	7. **F**	8. **p**	9. **SD**
10. ***cpmg***	11. **Total corr.**	12. 66	13. 66	14. 1	15.	16.	17. 1
	18. **Regression**	19. 30.40	20. 4	21. 7.60	22. 13.24	23. 7.45 e-8	24. 2.76
	25. **Residual**	26. 35.60	27. 62	28. 0.57	29.	30.	31. 0.76
32. ***led***	33. **Total corr.**	34. 66	35. 66	36. 1	37.	38.	39. 1
	40. **Regression**	41. 31.15	42. 4	43. 7.79	44. 13.86	45. 3.94e-8	46. 2.79
	47. **Residual**	48. 34.85	49. 62	50. 0.56	51.	52.	53. 0.75

SS- Sum of Squares; **DF**- Degrees of Freedom; **MS**- Mean Squares; **F**- F-test; **p**- p-value; **SD**- standard deviation
